# Modulation of melanoma cell phospholipid metabolism in response to heat shock protein 90 inhibition

**DOI:** 10.18632/oncotarget.125

**Published:** 2010-07-09

**Authors:** Mounia Beloueche-Babari, Vaitha Arunan, L. Elizabeth Jackson, Nina Perusinghe, Swee Y. Sharp, Paul Workman, Martin O. Leach

**Affiliations:** ^1^Cancer Research UK and EPSRC Cancer Imaging Centre, Section of Magnetic Resonance, The Institute of Cancer Research and The Royal Marsden NHS Foundation Trust, Sutton, Surrey SM2 5PT, United Kingdom; ^2^Cancer Research UK Centre for Cancer Therapeutics, The Institute of Cancer Research, Sutton, Surrey SM2 5NG, United Kingdom

**Keywords:** Hsp90, melanoma, metabolism, phospholipid, MRS

## Abstract

Molecular chaperone heat shock protein 90 (Hsp90) inhibitors are promising targeted cancer therapeutic drugs, with the advantage that they deplete multiple oncogenic client proteins and modulate all the classical hallmarks of cancer. They are now in clinical trial and show potential for activity in melanoma and other malignancies. Here we explore the metabolic response to Hsp90 inhibition in human melanoma cells using magnetic resonance spectroscopy. We show that, concomitant with growth inhibition and re-differentiation, Hsp90 inhibition in human melanoma cells is associated with increased glycerophosphocholine content. This was seen with both the clinical geldanamycin-based Hsp90 drug 17-AAG and the structurally dissimilar Hsp90 inhibitor CCT018159. The effect was noted in both BRAF mutant SKMEL28 and BRAF wildtype CHL-1 melanoma cells. Elevated content of the -CH2+CH3 fatty acyl chains and cytoplasmic mobile lipid droplets was also observed in 17-AAG-treated SKMEL28 cells. Importantly, the phospholipase A2 inhibitor bromoenol lactone prevented the rise in glycerophosphocholine seen with 17-AAG, suggesting a role for phospholipase A2 activation in the Hsp90 inhibitor-induced metabolic response. Our findings provide a basis for using metabolic changes as non-invasive indicators of Hsp90 inhibition and potentially as biomarkers of anticancer activity with Hsp90 drugs in malignant melanoma and possibly in other cancers.

## INTRODUCTION

Oncogenic transformation is associated with alterations in cellular metabolism that are necessary to sustain rapid proliferation and survival in the hostile tumor microenvironment. These changes, also referred to as ‘metabolic transformation’, include increased rates of aerobic glycolysis, protein and lipid synthesis [1-3].

A technique that has been especially useful in translating metabolic studies from cell models to humans is magnetic resonance spectroscopy (MRS), a non-invasive methodology for studying a wide array of cellular metabolites. MRS studies have shown increases in levels of membrane phospholipid metabolism intermediates including phosphocholine (PC), phosphoethanolamine (PE), glycerophosphocholine (GPC) and glycerophosphoethanolamine (GPE) in cancer compared to normal tissues [4, 5]. These changes were recorded in cells, animal models and patients, suggesting their value as potential markers of malignant disease and response to therapy [4, 6].

It is now widely accepted that these metabolic alterations occur as a result of the modulation of enzymes mediating phospholipid turnover, such as phospholipases A, C and D (catabolic pathway) and choline kinase and choline transport into cells (anabolic pathway) which have all been reported to be altered in malignancy as a result of oncoprotein activation [7, 8]. Furthermore, inhibition of oncogenic signaling by molecularly targeted anticancer agents produces changes in cellular phospholipid metabolism that are consistent with the induced molecular and cellular effects of the therapy (reviewed in ref [9]). Therefore, probing the metabolic effects of treatment with targeted agents should not only help to characterize the interactions between oncogenic signaling and tumor cell metabolism, but also provide a potential tool for monitoring the action of molecularly targeted drugs.

Among the many targeted agents designed to block the activity of oncogenic proteins, inhibitors of the heat shock protein 90 (Hsp90) molecular chaperone are of special interest. These agents cause the simultaneous depletion of many oncogenic chaperone ‘client’ proteins, thus leading to the parallel blockade of several oncogenic pathways and multiple key cancer cell functions including proliferation, invasion and angiogenesis [10-12]. Several Hsp90 inhibitors have been identified, including the benzoquinone ansamycin derivative 17-allylamino-17-demethoxygeldanamycin (17-AAG, tanespimicin), as well as the prototype of the pyrazole/isoxazole resorcinol class CCT018159 which is a precursor of the resorcinylic isoxazole amide clinical candidate NVP-AUY922 [13-16].

17-AAG has shown promising antitumor activity in numerous preclinical models, and is currently undergoing clinical testing with evidence of activity in trastuzumab-refractory breast cancer [17-19]. In addition, preclinical studies indicated that 17-AAG has therapeutic activity against melanoma cells, most likely via effects on the RAF family of oncoproteins [20, 21]. Early clinical studies revealed evidence of Hsp90 target modulation and some signs of biological activity (stable disease) in melanoma patients that may potentially be associated with the presence of BRAF or NRAS mutation [17, 22]. Interestingly, mutant BRAF, which is present in almost 70% of all melanomas, shows marked dependency on Hsp90 and is degraded by 17-AAG treatment, while WT BRAF activated by NRAS is also Hsp90 dependent [20]. Furthermore, melanoma cells, regardless of their BRAF or NRAS mutation status, are sensitive to the growth inhibitory effects of 17-AAG (21), consistent with the action of Hsp90 inhibitors on multiple oncoproteins, including CRAF. A recent phase II clinical study reported a lack of activity of 17-AAG in melanoma which may be attributed to the treatment dose and schedule not achieving sustained depletion of BRAF/CRAF kinases [23]. The authors recommended that future trials in melanoma should focus on more potent Hsp90 inhibitors or a 17-AAG formulation that can be administered chronically for a more prolonged suppression of the extracellular signal-regulated kinase1/2 (ERK1/2) mitogen activated protein kinase (MAPK) pathway.

In this study, we wished to study in more depth the effects of Hsp90 inhibitors in human melanoma cells, and in particular to explore the effects of Hsp90 inhibition on cell metabolism, which may potentially provide a means for further characterizing the anticancer activity of these agents in the clinical setting.

Membrane phospholipid metabolism is tightly linked to cell proliferation and survival [24, 25] and is altered with malignancy and following anticancer treatment [9, 26]. In melanoma, choline-containing derivatives of phospholipid metabolism show distinct profiles during growth inhibition by chemotherapy versus subsequent adaption to treatment [27]. This suggests that phospholipid metabolism may be a mediator of cell response/adaptation to treatment [27].

Using MRS, we have previously shown that inhibition of Hsp90 with 17-AAG in human colon carcinoma cells correlated with alterations in phospholipid metabolism; an elevation in PC and GPC levels *in vitro*, and PC+PE levels *in vivo* [28]. In other studies, however, 17-AAG treatment caused a decline in total choline levels (comprised of choline, PC and GPC) in prostate cancer xenografts [29] and the rates of radiolabelled-choline uptake and phosphorylation in human colon cancer cells which would be expected to produce a decrease in cellular PC [30]. The basis for these apparent discrepancies is unclear but could relate to variations in the genetic profiles and drivers of the various cancer cell lines studied or to differing downstream cellular effects of the therapy, such as differentiation or inhibition of growth versus induction of apoptosis [13. 31].

Here we explore the metabolic consequences of Hsp90 inhibition in human melanoma cells. Our aims were to characterize any metabolic changes in relation to the cellular and molecular effects induced post- Hsp90 inhibition, and to explore the mechanistic basis that could give rise to them. Our findings show that inhibition of Hsp90 in human melanoma cells with 17-AAG and CCT018159 is associated with reduced proliferation and induction of cell differentiation. These effects correlated with an elevation in cellular GPC and cytoplasmic lipid droplets which may be associated with activation of calcium-independent phospholipase A2 (iPLA2).

## MATERIALS & METHODS

### Cell culture

Human malignant melanoma SKMEL28 cells (with V600E mutant BRAF, WT NRAS) were obtained from ATCC, and CHL-1 cells (with WT BRAF, WT NRAS) were a gift from Prof Richard Marais (Institute of Cancer Research, London). Both cell lines were cultivated in DMEM containing 10% (v/v) heat inactivated fetal calf serum, 100 U/ml penicillin and 100 μg/ml streptomycin (Life Technologies; Paisley, UK) and monthly screened for mycoplasma.

### Analysis of cell growth inhibition, cell volume and cell cycle profiles

Cell counts and diameter measurements were performed on a Beckman Coulter Vi-Cell® Cell Viability Analyzer. The impact of the Hsp90 inhibitors 17-AAG (Alexis; Exeter, UK) and CCT018159 (Calbiochem; Nottingham, UK) on cell proliferation was assessed using the sulforhodamine B (SRB) assay following a 144h exposure to a range of drug concentrations as previously described [47]. The effect of Hsp90 inhibition on cell cycle profiles was assessed by flow cytometry using propidium iodide staining and standard procedures as previously described [47].

### Western blotting

The level of client protein expression following Hsp90 inhibition was assessed by Western blotting as previously described [47]. The primary antibodies used were anti-Hsp70 (Stressgen Bioreargents; Michigan, USA), anti-CRAF, anti-BRAF, anti-CDK4 (Santa Cruz Biotechnology; Santa Cruz, CA, USA), anti- total and phosphorylated cytosolic phospholipase A2 (cPLA2, Cell Signaling Technology; Danvers, MA, USA), antityrosinase and anti-gp100 (Abcam; Cambridge, UK) and anti-GAPDH (Chemicon; Hampshire, UK) antibodies. The secondary antibodies used were antimouse for BRAF, Hsp70 and GAPDH and anti-rabbit for CRAF, CDK4, cPLA2, phospho-cPLA2, tyrosinase and gp100 (GE Healthcare Life Sciences; Buckinghamshire, UK).

### Analysis of cell morphology

Bright field images of control and treated cells were acquired on a Zeiss Axiovert inverted light microscope (Carl Zeiss Ltd.; Hertfordshire, UK) connected to a Cool SNAP Pro Color digital camera (Media Cybernetics; Bethesda, MD, USA), and cells visualized using Image Pro Plus software (Media Cybernetics) version 6.2.0424.

### Cell treatment and extraction for MRS analyses

Logarithmically growing SKMEL28 and CHL-1 cells were treated with 100 nM or 39 nM 17-AAG respectively for 48h to achieve modulation of Hsp90 client proteins and a ca. 50% reduction in cell counts. SKMEL28 cells were further treated with 15 îM CCT018159 for 48h. Control cells were exposed to DMSO at a concentration of ≤0.1%. In subsequent experiments, 17-AAG treated cells were co-incubated with 20 îM of the iPLA2 specific inhibitor BEL (Sigma-Aldrich) for the last 24h of treatment.

At the end of each experiment cells were washed in cold saline and extracted in equal volumes of cold methanol, chloroform and water. Lyophilised samples of the water-soluble phase were reconstituted in 540 μl of a D_2_O solution containing 0.075% (w/v) 3- (trimethylsilyl)propionic-*2,2,3,3-d*_4_ acid as internal reference for analysis by ^1^H MRS. Following acquisition of 1H spectra, the sample volume was topped up to 600 μl with a D_2_O solution containing EDTA and methylenediphosphonic acid (internal standard) to a final concentration of 10 mM and 0.43 mM respectively at pH 8.2 for analysis by ^31^P MRS. The lipid fraction of cell extracts was reconstituted in CDCl3 containing 0.56 mM trimethyl silane as an internal standard.

### MR spectroscopy measurements

^1^H MR spectra were acquired at room temperature on a 500 MHz Bruker spectrometer using a 30° flip angle, a 1s repetition delay (RD), a spectral width of 13 ppm and 64 K data points under conditions of water signal suppression for aqueous samples. 31P MR spectra were acquired using power gated composite pulse ^1^H decoupling, a 30° flip angle, a 1s RD, a spectral width of 100 ppm and 32 K data points. Spectra were processed using MestRe-C version 2.3. (University of Santiago de Compostela, Spain), applying a 1Hz line broadening for 31P spectra. Metabolite content was determined by peak integration, normalized relative to the internal standard and corrected for cell number and volume and for saturation in the case of the ^31^P MR spectra.

### Mobile lipid staining and confocal microscopy

For the detection of neutral mobile lipids, SKMEL28 cells were grown on glass coverslips in four-well plates and treated with vehicle or 17-AAG as indicated above. At the end of the incubation, cells were washed in PBS then fixed in 4% paraformaldehyde followed by incubation in a 1î g/ml solution of Nile red (Sigma-Aldrich) for 15min. Cells were then washed three times in a PBS solution containing the nuclear stain Topro-3 (Molecular Probes; Paisley, UK), then mounted on a slide. Fluorescent images were acquired on a Leica SP1 confocal laser scanning microscope using 488 nm and 633 nm excitation, and 530 nm and 670-720 nm emission wavelengths for Nile red and Topro-3 respectively. Cells were visualized using the Leica Confocal software version 2.61. Quantitation of the number of lipid droplets per cell was performed using ImageJ (National Institutes of Health, USA) version 1.42q and analysis of a minimum of 15 cells per image.

### Statistical Analysis

The statistical significance of the results was assessed using an unpaired 2-tailed Student t-test with p values ≤ 0.05 considered to be significant. Data are represented as the mean ± standard deviation.

## RESULTS

### Hsp90 inhibition induces growth arrest and differentiation concomitant with depletion of oncogenic client proteins

SRB assay of growth inhibition following treatment with 17-AAG indicated that the drug reduced cell proliferation in the two human melanoma cell lines studied, with GI50 values of 21±5 nM for the BRAF mutant SKMEL28 cells and 13±2 nM for the BRAF WT CHL-1 cells. Thus 17-AAG has broadly similar potency in the two melanoma cell lines. This is in agreement with findings from previous studies in larger melanoma cell line panels showing no dependence of sensitivity to 17-AAG on BRAF mutation status [20, 21].

**Fig. 1:**
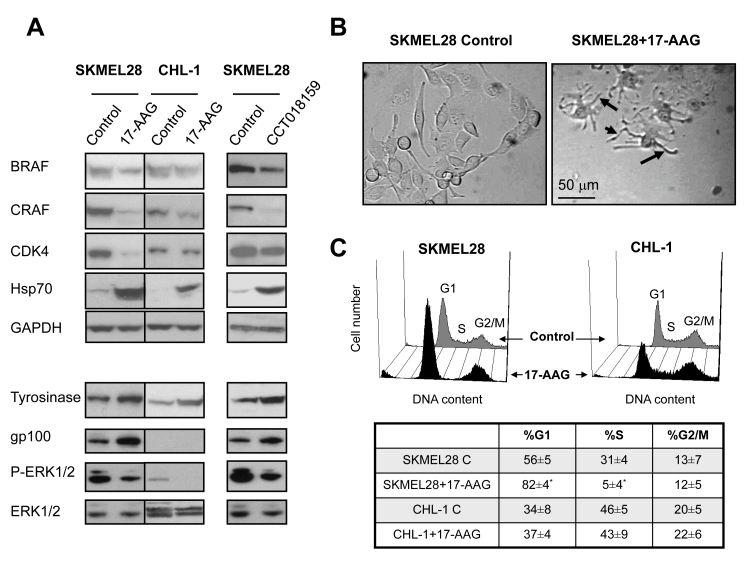
The effect of Hsp90 inhibitors on protein expression, cell morphology and cell cycle profiles in human melanoma cell lines. A) Western blot analysis showing the expression of Hsp90 client proteins, Hsp70 (top panel), phospho-ERK1/2, tyrosinase and gp100 in cells treated with 17-AAG (SKMEL28 and CHL-1), and CCT018159 (SKMEL28) for 48h at the concentrations indicated in the text. B) Bright field microscopy images showing the increased dendritic morphology (black arrows point to dendrites) of SKMEL28 cells following exposure to 100nM 17-AAG treatment for 48h. C) DNA histograms and a table showing cell cycle distributions in SKMEL28 and CHL-1 cells following exposure to vehicle or 17-AAG (100nM and 39nM respectively) for 48h. ^*^ p≤0.002.

Western blotting demonstrated the expected molecular signature of Hsp90 inhibition in both melanoma cell lines as shown by depletion of the client proteins CRAF, BRAF and CDK4 and induction of Hsp70 following exposure to 17-AAG, thus confirming inhibition of Hsp90 under the conditions used in our experiments (Figure 1A, top panel). To confirm that the effects were not restricted to one chemotype, the resorcinylic pyrazole Hsp90 inhibitor CCT018159 [16, 32] also inhibited growth in SKMEL28 cells with a GI50 value of 3.4±1.3 îM and induced similar molecular effects as 17-AAG in this melanoma line (Figure 1A).

Of interest, inhibition of Hsp90 resulted in induction of differentiation in the melanoma lines used, as shown by the characteristic increase in the dendritic cell morphology and expression of the melanocyte lineage marker tyrosinase in both CHL-1 and SKMEL28 cells (Figure 1A, lower panel and B). The levels of gp100, another melanoma differentiation marker, were undetectable in CHL-1 cells but significantly induced post-17-AAG treatment in SKMEL28 cells. Basal phospho-ERK1/2 levels were much lower in CHL-1 compared to SKMEL28 cells but decreased substantially post-exposure to 17-AAG in both cell lines and to CCT018159 in SKMEL28 cells (Figure 1A lower panel) consistent with reduced ERK1/2 MAPK signaling post Hsp90 inhibition.

As shown in Figure 1C, flow cytometry analysis of cell cycle profiles indicated that at the concentrations and treatment durations used, 17-AAG caused an increase in the G1 cell phase of the cell cycle and a corresponding reduction in the S phase population in SKMEL28 cells (n=3, p≤0.002). There was a small increase in the G1 cell population and a reduction in the S phase fraction in CHL-1 cells exposed to 17-AAG but these effects were not statistically significant (n=5, p≥0.27).

### Hsp90 inhibition leads to altered cellular choline phospholipid metabolism

Next, to evaluate cellular choline phospholipid metabolism following Hsp90 inhibition, we analyzed the ^1^H and ^31^P MR spectra of the aqueous metabolites from control and Hsp90 inhibitor-treated melanoma cells.

**Fig. 2:**
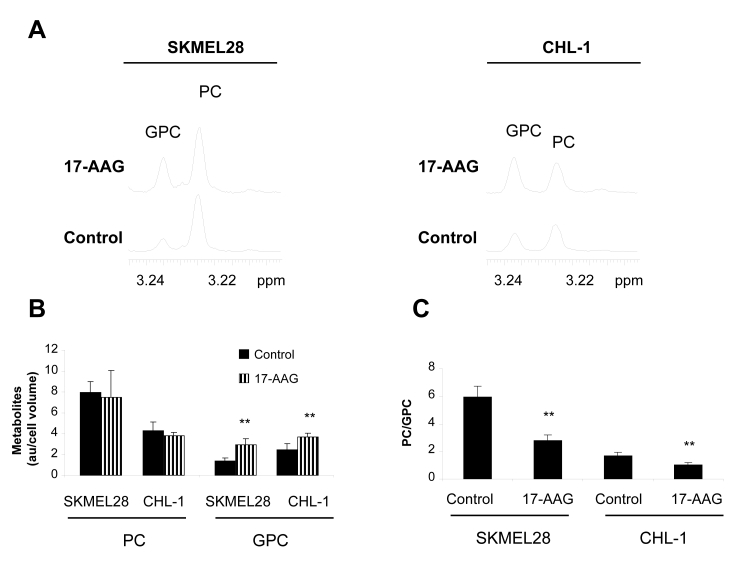
The effect of the Hsp90 inhibitor 17-AAG on ^1^H MRS detectable cellular choline metabolites in human melanoma cell lines. A) ^1^H MR spectra from the aqueous phase of cell extracts showing the choline region in control and 17-AAG treated SKMEL28 (left) and CHL-1 (right) cells. B) A histogram showing increased GPC content following 17-AAG treatment in SKMEL28 and CHL-1 cells while PC levels remain unchanged. C) A histogram showing decreased PC/GPC in 17-AAG treated SKMEL28 and CHL-1 cells relative to their vehicle-treated controls. **p<0.01.

^1^H MRS analysis indicated that the most prominent and consistent effect observed following 17-AAG treatment was a rise in GPC levels of up to 2.5-fold in SKMEL28 and CHL-1 cells (Figure 2A and B). PC levels remained unchanged in both cell lines (p≥0.35). Consequently, the PC/GPC ratio decreased by half in both SKMEL28 and CHL-1 cells (Figure 2C). Similar effects were also recorded by 31P MRS as summarized in Table 1.

**Table 1:** The effect of the Hsp90 inhibitor 17-AAG on the ^31^P-containing water soluble metabolites in SKMEL28 and CHL-1 human melanoma cells.

Metabolites (mM)	SKMEL28 (n=4)			CHL-1 (n=5)		
	Control	17-AAG	*p*	Control	17-AAG	*p*
**PC**	19.9±1.3	17.1±8.1	*0.69*	11.3±3.5	9.5±2.9	*0.38*
**GPC**	2.9±1.6	7.4±2.8*	*0.013*	6.7±1.8	9.6±1.6*	*0.015*
**GPE**	1.2±1.1	2.4±0.3	*0.18*	1.5±0.3	1.3±0.4	*0.63*
**NTP**	9.4±0.8	6.7±1.2*	*0.006*	8.6±2.4	8.1±2.8	*0.82*
NOTE: Metabolite levels represent average concentration in control or treated cells expressed as the mean ± SD. * Statistically significant difference (p<0.05, t-test) between control and treated cells.						

^1^H MRS analyses also indicated that SKMEL28 cells exhibited similar metabolic profiles to those seen with 17-AAG when exposed to the alternative chemotype Hsp90 inhibitor CCT018159, with the GPC content increasing to 240±65% relative to the controls (n=5, p=0.008). There was a trend towards an increase in PC content (152±46%) although this did not reach statistical significance (p=0.067). The PC/GPC ratio was reduced to 67±23% relative to vehicle treated controls (p=0.03).

### Hsp90 inhibition correlates with increased fatty acid signals and cytoplasmic lipid droplets

**Fig. 3:**
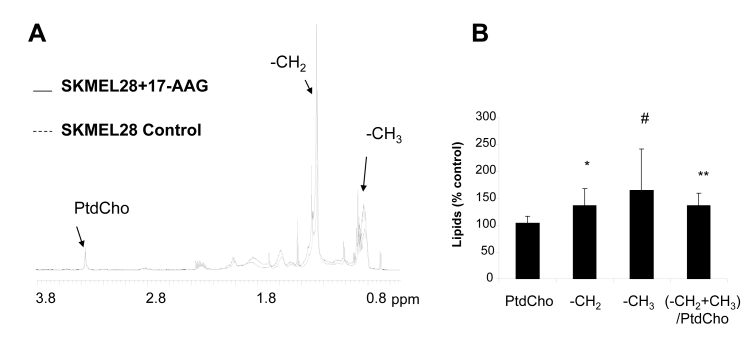
^1^H analysis of lipid metabolites in SKMEL28 human melanoma cells exposed to 17-AAG. A) Representative ^1^H MR spectra from the lipid fraction of control and 17-AAG-treated SKMEL28 cell extracts displaying the 0.5-3.8ppm region. B) A summary showing increased fatty acid signal intensities following exposure to 17-AAG. * p<0.05, ** p<0.01, # p=0.07.

GPC is formed following the breakdown of the membrane phospholipid phosphatidylcholine (PtdCho) via PLA2 and subsequently via lysophospholipase, leading to simultaneous release of free fatty acids (25). To investigate the basis for the rise in GPC, we analyzed the lipid metabolites of 17-AAG-treated SKMEL28 cells by ^1^H MRS. This revealed no differences in the levels of PtdCho (104±13%; n=7, p=0.4) but the levels of the -CH_2_ free fatty acyl chains increased reproducibly by 1.35-fold relative to the control (p=0.02). The content of the −CH_3_ chains and the poly-unsaturated fatty acid signals at 5.3 ppm was also increased by 1.65-and 1.3-fold respectively, although this did not reach statistical significance (n=7, p=0.07 and p=0.14 respectively). The ratio of – (CH_3_+CH_2_)/PtdCho increased by 1.35-fold relative to the control (p=0.0055) (Figure 3A and B).

**Fig. 4:**
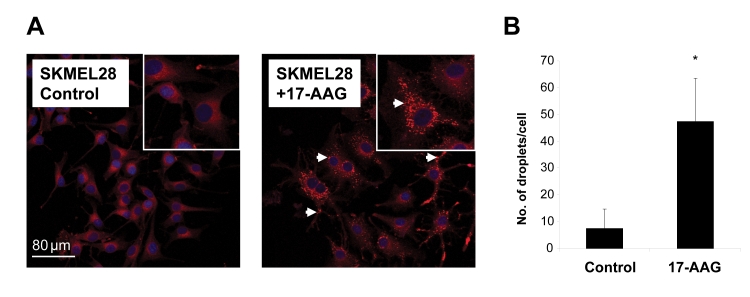
Changes in cytoplasmic lipid droplets in SKMEL28 human melanoma cells following 17-AAG treatment. A) Confocal microscopy images of control and 17-AAG-treated SKMEL28 cells stained with Nile red (procedure described in Materials and Methods) showing staining intensity from cytoplasmic lipid droplets (white arrows). B) Quantitative analysis of the number of cytoplasmic lipid droplets in control and 17-AAG-treated cells. *p=0.033.

To further characterize the changes in lipids observed by MRS, we performed Nile red staining on SKMEL28 cells following exposure to 17-AAG. Analysis by confocal microscopy revealed an increase in the staining originating from cytoplasmic mobile lipid droplets following treatment with 17-AAG compared to vehicle treated control cells (Figure 4A). Quantitation showed an increase in the number of lipid droplets by ~6.5-fold compared to the control (n=3, p=0.033). This result corroborates the ^1^H MRS-observed increase in fatty acyl chain signals post 17-AAG treatment and indicates that these are likely to be present within cytoplasmic mobile lipid stores.

### The metabolic effects of Hsp90 inhibition may be associated with calcium-independent PLA2 activation

**Fig. 5:**
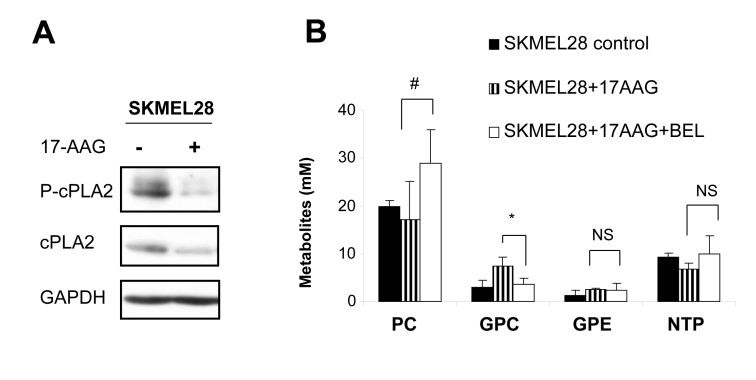
The involvement of PLA2 activity in mediating the metabolic effects of 17-AAG. A) Western blots showing decreased expression of cPLA2 and phospho-cPLA2 in SKMEL28 following exposure to 17-AAG. B) A histogram showing that the specific iPLA2 inhibitor BEL is able to prevent the rise in GPC after 17-AAG treatment, with no significant effect on the other metabolites (as detected by ^31^P MRS analysis). ^*^ p=0.01, #p=0.076, NS p≥0.37.

The increase in GPC coupled with the rise in mobile lipids induced by 17-AAG pointed to activation of PLA2 following Hsp90 inhibition. To confirm this, we assessed the effect of 17-AAG on the level of expression and activation (by phosphorylation) of cytosolic calcium-dependent PLA2 (cPLA2), a widely studied and well characterized member of the PLA2 family (34). Interestingly, Western blotting showed a reduction in the expression of cPLA2 together with a marked decline in the levels of phospho-cPLA2 in SKMEL28 cells following exposure to 17-AAG, indicating reduced activity of the enzyme (Figure 5A). Therefore, it is unlikely that the metabolic changes observed following treatment with 17-AAG could be related to cPLA2 activation.

We next tested the involvement of cytosolic calcium-independent PLA2 (iPLA2), another key regulator of membrane lipid metabolism [34], and assessed whether the effect on GPC could be reversed by co-treatment with the iPLA2 specific inhibitor BEL [34].

Western blotting showed that the levels of Hsp90 client protein depletion and differentiation marker induction were comparable in melanoma cells treated with 17-AAG+BEL versus 17-AAG alone (data not shown). In addition, cell counts per flask relative to the control were also similar (40±6% for 17-AAG and 34±14% for 17-AAG+BEL, p=0.46). MRS analysis indicated that, as shown in Figure 5B, co-incubation of SKMEL28 melanoma cells with 17AAG+BEL prevented the rise in GPC seen with 17-AAG alone (n=4, p=0.01). PC levels showed a slight increase in 17-AAG+BEL treated melanoma cells compared to cells treated with 17-AAG alone although this effect was not statistically significant (p=0.076). No significant alterations were noted in the levels of GPE or NTP in cells treated with 17-AAG+BEL versus 17-AAG on its own (p≥0.37, Figure 5B).

These observations suggest that the rise in GPC recorded with 17-AAG may be related to iPLA2 activation following Hsp90 inhibition.

## DISCUSSION

Studies using MRS have shown characteristic changes in choline phospholipid metabolism in tumor versus normal tissues, which extend from cell and animal models to humans. In particular, increases in levels of PC and the PC/GPC ratio have been reported to occur with increased malignant potential [7, 26]. This ‘metabolic signature’ has been linked to changes in enzyme activities governing the rate of choline transport and phosphorylation (e.g. choline kinase) as well as membrane phospholipid breakdown (PLA, C and D) which occur following oncogene activation and the acquisition of a malignant phenotype [7, 8, 35]. In a melanoma model, alterations in the phospholipid metabolic phenotype were observed during initial response to chemotherapy versus subsequent adaptation of tumors to treatment. Specifically during the growth inhibition phase, a rise in levels of PC+PE and GPC+GPE was recorded while the re-growth phase was characterized by an increase in PC+PE only. It is therefore plausible that changes in tumor phospholipid profiles could be implicated in tumor survival and adaptation to therapy [27].

In this study we evaluate the metabolic consequences that follow inhibition of Hsp90, a molecular chaperone involved in maintaining the stability, conformation and activatable state of several oncogenic proteins involved in the initiation and maintenance of the malignant phenotype [10-12]. We used the clinical Hsp90 inhibitor 17-AAG and the prototype resorcinylic pyrazole CCT018159 Hsp90 inhibitor in human melanoma cells to study the effects of inhibition of the molecular chaperone on phospholipid metabolism, and to explore the cellular and biochemical processes that give rise to them.

Two human melanoma cell lines were used: CHL-1 with WT BRAF/WT NRAS and SKMEL28 with V600E mutant BRAF/WT NRAS. We selected these lines because mutant BRAF is highly sensitive to Hsp90 inhibition although melanoma cells regardless of their BRAF status are also growth inhibited by agents such as 17-AAG, most likely because of depletion of CRAF as well as BRAF, in addition to effects on other client proteins [20, 21].

Growth inhibition assays on SKMEL28 and CHL-1 cells showed no major differences in the potency of 17-AAG between the two cell lines, consistent with previous studies in larger panels showing that mutant BRAF is not a predictive marker for sensitivity to Hsp90 inhibitors in human melanoma cell lines [20, 21].

Further assays were conducted to determine the time and drug concentration required to achieve the characteristic molecular signature of Hsp90 inhibition (reduction in CRAF, BRAF and CDK4 as representative client proteins together with induction of Hsp70) as well as to cause a decrease in cell counts to ~50% of controls. Interestingly, inhibition of Hsp90 with both 17-AAG and CCT018159 was associated with induction of cell differentiation as indicated by the increased expression of the melanocyte lineage markers tyrosinase and gp100 (involved in melanin synthesis and deposition) and the increase in the dendritic cell morphology [36]. These observations are in agreement with the previously reported effects of Hsp90 inhibition on cell differentiation status in human breast cancer cells [31].

We next examined the metabolic features characteristic of cells exhibiting the molecular and cellular effects of Hsp90 blockade. ^1^H and ^31^P MR analyses of extracts from SKMEL28 and CHL-1 cells indicated that 17-AAG treatment resulted in an increase in cellular levels of GPC in the two cell lines while PC levels remained unchanged. The PC/GPC ratio was also reduced in both cell lines. Similar changes in GPC and PC/GPC were also obtained with the more recently developed and structurally dissimilar Hsp90 inhibitor CCT018159 in SKMEL28 melanoma cells. Demonstration of the same effects with two different Hsp90 inhibitor chemotypes provides further support for a mechanism involving pharmacologic modulation of the molecular chaperone. This metabolic signature correlated with inhibition of Hsp90 and downstream growth arrest and induction of differentiation in both melanoma cell lines used here, suggesting that it is likely to be BRAF statusindependent.

Membrane phospholipid metabolism is regulated by several processes including the cell cycle, with net phospholipid accumulation observed during S phase [24]. In our experiments, Hsp90 inhibition caused a G1/S cell cycle arrest in SKMEL28 cells, while the effects in CHL-1 cells were not statistically significant; GPC, however, increased in both cases. Therefore it is unlikely that the rise in GPC observed post Hsp90 inhibitor treatment is related to an arrest in a particular phase of the cell cycle.

Previous work has shown increased GPC in cells exposed to apoptosis inducing agents [37]. Although Hsp90 inhibitors are mainly cytostatic, they can in some cases induce apoptosis, albeit to a limited extent due to the anti-apoptotic effects of induced heat shock proteins, such as Hsp72 and Hsc70 [12, 16, 38]. Our measurements were performed on adherent cells which were largely non-apoptotic as shown by flow cytometry (Figure 1C). Therefore it is unlikely that the changes reported here could be linked to overt apoptosis, although we cannot rule out the involvement of early processes leading up to apoptosis.

The decline in the PC/GPC ratio observed in every case is consistent with reduced malignant potential [26, 35] following treatment with Hsp90 inhibitors and indicates a degree of normalization in cellular phospholipid metabolism post-therapy.

As indicated above, GPC is produced following the hydrolysis of PtdCho via PLA2 and subsequently lysophospholipase, which results in simultaneous release of free fatty acids [25]. To explore the mechanistic basis for the rise in GPC detected here, we assessed the levels of free fatty acid chains post Hsp90 inhibition. ^1^H MRS analysis showed an increase in the −CH_2_+CH_3_ fatty acyl groups in 17-AAG-treated SKMEL28 cells concomitant with the accumulation in GPC. To better characterize the origin of these lipids, Nile red staining was performed. This showed that 17-AAG treatment resulted in an increase in the signals originating from cytoplasmic mobile lipid droplets, which are believed to contain the −CH_2_+CH_3_ fatty acyl chains detectable by MRS [39, 40]. Taken together these data support the involvement of a phospholipase A2 activity in the metabolic alterations observed following Hsp90 inhibition.

The PLA2 superfamily consists of many members, of which three main types are distinguishable: the cytosolic calcium-dependent (cPLA2), cytosolic calcium-independent (iPLA2), and the secreted (sPLA2) PLA2 [34]. To determine the particular isoform involved in the observed metabolic changes, we initially investigated the expression and status of activation (by phosphorylation) of the widely reported and well characterized cPLA2 using Western blotting. This analysis indicated that the levels of cPLA2 and phospho-cPLA2 (the active form) were markedly reduced upon exposure to 17-AAG (an effect that to our knowledge has not been previously reported). Therefore it is unlikely that this enzyme could be involved in the increased generation of GPC observed here. This result was not surprising since a previous study showed that cPLA2 is activated by mitogenic signaling (which, at least for ERK1/2, is inhibited by 17-AAG in this study) leading to arachidonic acid mobilization, necessary for the inflammatory response [41], in line with the reported anti-inflammatory effects of Hsp90 inhibition [42].

On the other hand, iPLA2 has been implicated in phospholipid remodeling and in GPC release to maintain cellular lipid homeostasis [43]. Due to the lack of good commercially available antibodies for iPLA2 (and sPLA2), we could not evaluate the expression of these enzymes following exposure to 17-AAG. Therefore, to assess if iPLA2 could be implicated in the rise in GPC, 17-AAG-treated SKMEL28 cells were co-incubated with BEL, a specific inhibitor of iPLA2 that has no activity against the other cellular PLA2s including cPLA2 and sPLA2 [34]. Our results show that BEL treatment preserved the molecular response of Hsp90 inhibition without further significant reduction of cell counts. More importantly co-exposure to BEL prevented the accumulation in GPC recorded with 17-AAG alone with no significant effect on PC, GPE or NTP. This finding indicates that the rise in GPC seen with Hsp90 inhibitors is likely to involve iPLA2 activation.

Despite the fact that BEL is specific for iPLA2 over the other PLA2 sub-types, it also inhibits the magnesium-dependent phosphatidic acid phosphatase, the enzyme that converts phosphatic acid into diacylglycerol, with similar potency [44]. However, as this activity is not involved in the synthesis of GPC, it is unlikely that the effects of BEL on GPC observed here could be linked to inhibition of enzymes other than iPLA2. We therefore conclude that activation of iPLA2, but not cPLA2, may be implicated in the accumulation of GPC content seen following Hsp90 inhibition. The observation that PtdCho levels were not altered in 17-AAG-treated cells despite the presumed up-regulation of iPLA2 activity is consistent with the tight control of membrane PtdCho homeostasis and implies the involvement of other rate limiting enzymes in regulating this process.

The exact molecular mechanism via which Hsp90 inhibition impacts on iPLA2 activity needs further investigation. One possibility may be through phosphorylation of p38 MAPK occurring following Hsp90 inhibition [45] which has been shown to activate iPLA2 [46]. Future work will establish whether such events could be responsible for the changes observed in our study.

We have shown that the rise in GPC was the most consistent change seen in SKMEL28 and CHL-1 melanoma cell lines, and was seen with both Hsp90 inhibitor chemotypes in SKMEL28 cells. This is in agreement with results from our previous work with 17-AAG showing increased GPC levels in human colon cancer cells [28]. However, and in contrast to our earlier findings from that study, we did not observe a significant effect on PC levels following Hsp90 inhibition in the human melanoma cell lines used here. Moreover, an *in vivo* study on prostate cancer xenografts has shown a reduction in total choline levels (comprising choline, PC and GPC) following administration of 17-AAG [29]; however, in that *in vivo* study there was significant apparent tumor heterogeneity following treatment with appearance of a potentially necrotic core, which may have altered the overall metabolite signature *in vivo*, and contributed to partial volume effects. The metabolic response in the absence of the tissue effects of treatment was not reported. We have previously noted metabolic changes *in vivo* that are proportional to the degree of volume response and to vascular changes [47]. The apparent discrepancy between the various results may indicate cell line and context-dependence and further investigation is required to define the basis for these differences in metabolic effects.

In summary, our data show that Hsp90 inhibition results in increased GPC and reduced PC/GPC in human melanoma cells. This is coupled with elevated fatty acid signals and cytoplasmic mobile lipid droplet content. The rise in GPC is reversed upon co-exposure to the iPLA2 specific inhibitor BEL, indicating the involvement of iPLA2 as a likely mechanism for the increase in the levels of this metabolite. This metabolic effect was concomitant with inhibition of oncogenic signal transduction and cell proliferation, and induction of differentiation in a manner that does not seem to be dependent on BRAF mutational status or on the particular cell cycle arrest profile induced in the melanoma cell lines studied here.

These findings offer further insights into the biochemical processes modulated by Hsp90 inhibition in human melanoma cells, and provide a basis for future studies investigating metabolic changes as potential indicators of the anticancer activity exerted by Hsp90 inhibitors in malignant melanoma.

## Abbreviations used:

BEL: Bromoenol lactone
ERK: Extracellular signal regulated kinase
GPC: Glycerophosphocholine
GPE: Glycerophosphoethanolmine
Hsp90: Heat shock protein 90
MAPK: Mitogen activated protein kinase
MRS: Magnetic resonance spectroscopy
NTP: Nucleotide triphosphate
PC: Phosphocholine
PE: Phosphoethanolamine
PLA2: Phospholipase A2
PtdCho: Phosphatidylcholine
SRB: Sulforhodamine B
